# Isolation and Genomic Characterization of a Proteobacterial Methanotroph Requiring Lanthanides

**DOI:** 10.1264/jsme2.ME19128

**Published:** 2020-02-08

**Authors:** Souichiro Kato, Motoko Takashino, Kensuke Igarashi, Wataru Kitagawa

**Affiliations:** 1 Bioproduction Research Institute, National Institute of Advanced Industrial Science and Technology (AIST), Sapporo, Japan; 2 Division of Applied Bioscience, Graduate School of Agriculture, Hokkaido University, Sapporo, Japan; 3 Computational Bio Big Data Open Innovation Laboratory (CBBD-OIL), AIST, Sapporo, Japan

**Keywords:** methanotroph, methanol dehydrogenase, lanthanides, isolation, comparative genomics

## Abstract

Although the bioavailability of rare earth elements (REEs, including scandium, yttrium, and 15 lanthanides) has not yet been examined in detail, methane-oxidizing bacteria (methanotrophs) were recently shown to harbor specific types of methanol dehydrogenases (XoxF-MDHs) that contain lanthanides in their active site, whereas their well-characterized counterparts (MxaF-MDHs) were Ca^2+^-dependent. However, lanthanide dependency in methanotrophs has not been demonstrated, except in acidic environments in which the solubility of lanthanides is high. We herein report the isolation of a lanthanide-dependent methanotroph from a circumneutral environment in which lanthanides only slightly dissolved. Methanotrophs were enriched and isolated from pond sediment using mineral medium supplemented with CaCl_2_ or REE chlorides. A methanotroph isolated from the cerium (Ce) chloride-supplemented culture, *Methylosinus* sp. strain Ce-a6, was clearly dependent on lanthanide. Strain Ce-a6 only required approximately 30 nM lanthanide chloride for its optimal growth and exhibited the ability to utilize insoluble lanthanide oxides, which may enable survival in circumneutral environments. Genome and gene expression analyses revealed that strain Ce-a6 lost the ability to produce functional MxaF-MDH, and this may have been due to a large-scale deletion around the *mxa* gene cluster. The present results provide evidence for lanthanide dependency as a novel survival strategy by methanotrophs in circumneutral environments.

Aerobic methane-oxidizing bacteria (methanotrophs) utilize methane as their sole carbon and energy source, and play a significant role in the global climate by contributing to reductions in the emission of methane into the atmosphere, the second most important greenhouse gas after carbon dioxide (Hanson and Hanson, 1996). Various environmental factors, including temperature, methane and oxygen concentrations, and the availability of some metals and nitrogenous compounds, have been shown to influence the community structure and total activity of methanotrophs (Semrau *et al.*, 2010). The availability of Cu has been intensively investigated as a key factor that affects the physiology and activity of methanotrophs because the first step in methane oxidation is performed by two different methane monooxygenases (MMOs): a Cu- and Fe-containing particulate enzyme (pMMO) with relatively high activity and substrate affinity, and an Fe-containing soluble enzyme (sMMO) (Semrau *et al.*, 2018).

Rare earth elements (REEs) form a chemically uniform group and include two non-lanthanides (scandium [_21_Sc] and yttrium [_39_Y]) and 15 lanthanides (lanthanum [_57_La] to lutetium [_71_Lu]). Despite their name, REEs are not very rare. The relative abundance of REEs in the Earth’s crust and also in soil and sediment particles is as high as 200 ppm, which is similar to those of some essential metals, such as Cu and Zn (Tyler, 2004). However, REEs have long been regarded as non-essential elements for living organisms. Specific types of bacterial methanol dehydrogenases (XoxF-MDHs) were recently found to contain certain lanthanides in their catalytic centre, whereas their well-characterized counterparts (MxaF-MDHs) were Ca^2+^-containing enzymes (Hibi *et al.*, 2011; Chistoserdova, 2016). Furthermore, recent studies revealed that some non-methylotrophic bacteria harbor lanthanide-dependent alcohol dehydrogenases (Wehrmann *et al.*, 2017). Almost all aerobic methanotrophs have both XoxF- and MxaF-MDHs (Keltjens *et al.*, 2014). The expression of these alternative MDHs is regulated by the availability of lanthanides. In the presence of lanthanides, methanotrophs preferentially utilize XoxF-MDHs, which appear to be catalytically superior to MxaF-MDHs, whereas MxaF-MDHs are dominantly expressed in the absence of lanthanides (Farhan Ul Haque *et al.*, 2015; Chistoserdova, 2016; Krause *et al.*, 2017). The only known exception is the thermoacidophilic methanotroph in the phylum Verrucomicrobia, *Methylacidiphilum fumariolicum* SolV (Pol *et al.*, 2007), which exclusively has XoxF-MDH and exhibits lanthanide dependency for growth (Pol *et al.*, 2014). This strain was isolated from volcanic mudpots (>70°C, pH<1) in which lanthanides were present at concentrations of 2‍–‍3‍ ‍μM, which were markedly higher than those in moderate ecosystems (generally sub-nanomolar) (Elderfield *et al.*, 1990; Shiller *et al.*, 2017).

Since lanthanides are abundant in soil and sediment particles and all methanotrophs reported to date have XoxF-MDH (Keltjens *et al.*, 2014), we assume the existence of lanthanide-dependent methanotrophs in moderate environments. This assumption is supported by previous findings on methanol-oxidizing bacteria (methylotrophs) that show lanthanide dependency (Skovran and Martinez-Gomez, 2015; Lv *et al.*, 2018). Furthermore, the presence of lanthanide-dependent methanotrophs in moderate environments has been deduced from biogeochemical and molecular ecological studies. Methanotrophic activities in marine environments correlated with the depletion of light lanthanides (Shiller *et al.*, 2017). The existence of methanotrophs exclusively containing XoxF-MDH was previously proposed by a metagenomic analysis on enrichment cultures derived from marine sediment (Vekeman *et al.*, 2016). However, lanthanide-dependent methanotrophs have not yet been isolated from moderate environments. The aims of the present study were to enrich and isolate mesophilic and neutralophilic methanotrophs requiring lanthanides from a moderate environment and to investigate their molecular backgrounds through a comparative genomic analysis.

## Materials and Methods

### Bacterial strains and culture conditions

*Methylosinus sporium* DSM17706^T^ and methanotrophs isolated in the present study were cultured in test tubes (26-mL capacity) filled with 5 mL of Ca-free inorganic basal medium comprising 5‍ ‍mM NaNO_3_, 2‍ ‍mM KH_2_PO_4_, 1‍ ‍mM MgCl_2_, 0.1‍ ‍mM Na_2_SO_4_, 20‍ ‍mM 4-(2-hydroxyethyl)-1-piperazineethanesulfonic acid (HEPES), and 10 mL L^–1^ each of a trace element solution (CaCl_2_ was omitted) and vitamin solution (Kato *et al.*, 2016). pH was adjusted to 7.0 using 6N KOH. The test tubes were sealed with butyl rubber stoppers and aluminium seals before autoclave sterilization. Depending on the experiment, CaCl_2_ and REE chlorides (final concentration of 20 μM each, unless otherwise stated) were supplemented from sterilized stock solutions after autoclaving. *M. sporium* DSM17706^T^ and strains Y-b3, La-a12, and Ce-a6 were routinely cultured in basal medium supplemented with CaCl_2_, YCl_3_, LaCl_3_, and CeCl_3_, respectively. Cultures were supplemented with methane (2 mL tube^–1^) or methanol (20‍ ‍mM) as the carbon and energy source and incubated at 30°C with shaking (180 rpm). In growth tests with different metal supplementation, precultured cells were washed twice with the basal medium before inoculation. Growth was monitored by measuring optical density at 600 nm (OD_600_). In the growth test with insoluble lanthanide, 1 g L^–1^ of Ce oxide particles (>99.9% purity; FUJIFILM Wako Pure Chemical) was supplemented into inorganic basal medium. Growth in the cultures supplemented with cerium oxide was assessed by measuring methanol concentrations using a high-performance liquid chromatograph (D-2000 LaChrom Elite HPLC system; HITACHI) as described previously (Kato *et al.*, 2014b) because Ce oxide particles affect OD_600_ values. After 4 days incubation, the culture supernatant was filtrated using a 0.2-μm pore filter membrane and subjected to an inductivity coupled plasma optical emission spectrometer (ICP-OES; ULTIMA2, Horiba) analysis to measure the concentrations of Ca and Ce ions. All culture experiments were conducted in triplicate. The Student’s *t*-test was used for statistical analyses.

### Enrichment cultures of methanotrophs

Enrichment cultures were performed under the same conditions as those described above. All cultures were supplemented with methane (2 mL tube^–1^) and CaCl_2_ or REE chlorides (20 μM each). Sediment samples were collected from 0 to 5 cm below the bottom of a shallow pond (Oono pond at Hokkaido University, Sapporo, Hokkaido, Japan, lat 43.0743, long 141.3419). Approximately 100‍ ‍μL of pond sediment slurry (containing both pond water and sediment soil) was inoculated as the microbial source. Growth was monitored by measuring the partial pressure of methane in the gas phase using a gas chromatograph (GC-2014; Shimadzu) as described previously (Kato *et al.*, 2014a). Two hundred microliters of enrichment cultures was transferred into fresh medium when >90% of methane was consumed. After at least five transfers, enrichment cultures were subjected to a microbial community analysis and the isolation of methanotrophs.

### Microbial community analysis

A clone library analysis targeting partial 16S rRNA genes was performed as described previously (Kato *et al.*, 2010). Genomic DNA was extracted using the FAST DNA Spin Kit for soil (MP Biomedicals) according to the manufacturer’s instructions. Partial 16S rRNA gene fragments were amplified by PCR using the primer pair 515′F/805R (Hugerth *et al.*, 2014) as described previously (Kato *et al.*, 2010). PCR products were purified using a QIAquick PCR Purification Kit (Qiagen), ligated into the pGEM-T Easy Vector (Promega), and cloned into *Escherichia coli* JM109 competent cells (Promega). The sequences of the cloned PCR products were elucidated at the Biomedical Center, TAKARA Bio. The sequences obtained were assigned to operational taxonomic units (OTUs) using the BLASTClust program (Altschul *et al.*, 1997) with a cut-off value of 98% sequence identity.

### Isolation of methanotrophs

Enrichment cultures were serially diluted with Ca-free inorganic basal medium and spread onto the same medium supplemented with either CaCl_2_ or REE chlorides and solidified with 1.5% (w/v) agar. The inoculated plates were incubated at 30°C in an AnaeroPack pouch bag (Mitsubishi Gas Chemical) filled with methane and air at a ratio of approximately 1:1. The colonies that formed were further purified by repetitive plating at least 3 times. The purity of the isolates was confirmed by observations under a microscope (ProvisAX70; Olympus). The methanotroph strains isolated in the present study, *Methylosinus* sp. Ce-a6, *Methylocystis* sp. La-a12, and *Methylocystis* sp. Y-b3, were deposited to the Japan Collection of Microorganisms at the RIKEN Bioresource Center (RIKEN-BRC JCM) under the culture collection accession numbers JCM 32771, JCM 32772, and JCM 32774, respectively.

### Phylogenetic analysis of isolates

Almost the full length of the 16S rRNA gene sequence was elucidated by the direct sequencing of PCR products with the primer pair 27F/1492R, as described previously (Kato *et al.*, 2004). The isolated strains were classified into phylotypes with a cut-off value of 98% sequence identity using the BLASTClust program (Altschul *et al.*, 1997). The closest relatives of the representative strains of each phylotype were inferred using the BLAST program (Altschul *et al.*, 1997). Phylogenetic trees were constructed by the neighbour-joining method (Saitou and Nei, 1987) using the program MEGA ver. 7 (Tamura *et al.*, 2013). To evaluate the robustness of the inferred trees, the bootstrap resampling method (Felsenstein, 1985) was used with 1,000 replicates.

### Draft genome analysis

The genomic DNAs of strain Ce-a6 and *M. sporium* DSM17706^T^ were isolated by the conventional method using lysozyme, protease K, and SDS for cell lysis, followed by phenol/chloroform extraction. Extracted DNA was used to generate Illumina shotgun paired-end (2×101 bp) sequence libraries, which were sequenced with an Illumina HiSeq 2500 platform (Illumina). The reads obtained were quality trimmed with Trimmomatic v0.33 (Bolger *et al.*, 2014) and assembled using SPAdes v3.10.1 (Bankevich *et al.*, 2012). The assembled contigs were annotated using Prokka v1.12 (Seemann, 2014).

### Quantitative RT-PCR (qRT-PCR)

Strain Ce-a6 was cultured in inorganic basal medium in the presence and absence of CaCl_2_ and CeCl_3_ (final 20 μM) with methane as the sole energy and carbon source until the mid-log phase. Total RNA was isolated from strain Ce-a6 cells using a bead-beating method as described previously (Kato *et al.*, 2014a). Specific primers targeting *xoxF* (Cea6-xoxF-1402f; 5′-ACC AAC ATG GGC AAT TTC AT-3′, Cea6-xoxF-1600r; 5′-TGC CCG ACG GAG TCT TAT AC-3′), truncated *mxaF* (Cea6-mxaF-937f; 5′-GAA GCC AAG TTC GGC TAT CA-3′, Cea6-mxaF-1137r; 5′-GAC CGT CTC GTC GAT CTT GT-3′), and *pmoA1* (Cea6-pmoA1-424f; 5′-CTG TCG GGC TCC TAT GTG AT-3′, Cea6-pmoA1-618r; 5′-CTC GAC CAT GCG GAT GTA TT-3′) of strain Ce-a6 were designed with Primer3 software (Untergasser *et al.*, 2012). A quantitative gene expression analysis based on one-step real-time RT-PCR was performed using the RNA-direct SYBR Green Realtime PCR Master Mix (Toyobo) and the Mx3000P System (Stratagene) as described previously (Kato *et al.*, 2014a). The gene for particulate methane monooxygenase subunit A (*pmoA1*) was used to normalize expression values because a previous study confirmed that the expression of *pmoA* genes was constitutive and not markedly affected under most conditions (including different lanthanide concentrations) other than different Cu concentrations for many methanotrophs (including *M. trichosporium*, a close relative of strain Ce-a6) (Farhan Ul Haque *et al.*, 2015).

### Nucleotide sequence accession numbers

The nucleotide sequence data obtained from the clone library analysis and the methanotrophs isolated in the present study have been submitted to the DNA Data Bank of Japan (DDBJ) under accession numbers LC380944–LC380991. Draft genome data for strain Ce-a6 and *M. sporium* DSM17706^T^ were deposited in DDBJ under accession numbers BGJX01000001–BGJX01000119 and BGJY01000001–BGJY01000114, respectively.

## Results and Discussion

### Enrichment culture of methanotrophs in the presence of different REEs

Since lanthanides function as a substitute for Ca, we prepared Ca-free inorganic basal medium and added 20 μM of chlorides of either Ca, a non-lanthanide REE (Sc or Y), or a lanthanide (La, cerium [Ce], neodymium [Nd], or dysprosium [Dy]). It is important to note that the basal medium contained a small amount of Ca^2+^ (~2 μM) possibly derived from impurities. Enrichment cultures were set up using methane as the sole carbon and energy source. The sediment of a small pond (Oono pond, Sapporo, Japan) was selected as a typical freshwater environment and was used as the microbial source. Pond water was circumneutral (pH 7.6), had a low ion strength (electrical conductivity: 15.8 S m^–1^), and was oligotrophic (chemical oxygen demand: <2 mg L^–1^) (Zhang and Ishii, 2018). Pond water contained approximately 63 μM of Ca^2+^, while the concentration of REE ions was under detection limit (<10 nM).

**Fig. 1.**
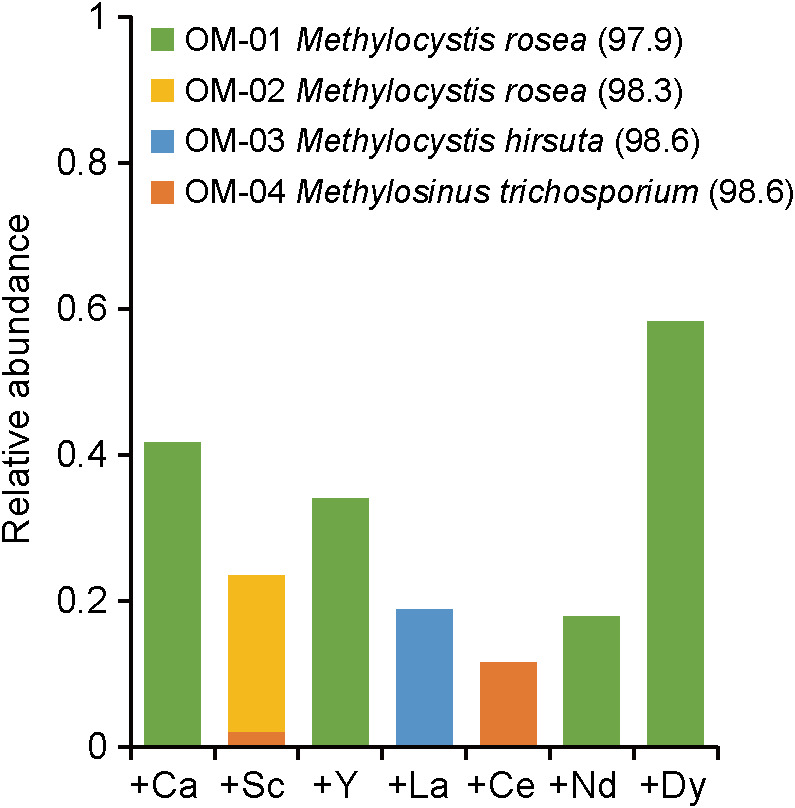
Effects of rare earth elements (REEs) on methanotrophic microbial communities derived from pond sediment. A clone library analysis targeting 16S rRNA genes was conducted for methanotrophic communities enriched from pond sediment in mineral medium supplemented with 20 μM of chlorides of either Ca, non-lanthanide REEs (Sc or Y), or lanthanides (La, Ce, Nd, or Dy). Only operational taxonomic units (OTUs) classified as methanotrophs are shown (see Fig. S1 for the distribution of non-methanotroph OTUs). OTU numbers and their closest relatives (sequence identity, %) are shown in the legend.

The consumption of methane and growth of microorganisms were observed in all enrichment cultures regardless of the supplemented metal species. After the enrichment cultures had been subcultured at least five times, we performed a clone library analysis targeting 16S rRNA genes to define the dominant methanotroph species in each enrichment. Among the 48 OTUs identified (Table S1), 4 were classified as methanotrophs in the family Methylocystaceae. The dominant methanotroph OTUs differed depending on the supplemented metal species (Fig. 1). In the +Ca, +Y, +Nd, or +Dy enrichments, OTU OM-01 was the only methanotroph OTU. In contrast, specific methanotroph OTUs, namely OM-02, -03, and -04, predominated in the +Sc, +La, and +Ce enrichments, respectively.

Although methane was supplied as the sole substrate, the relative abundance of methanotrophs in the enrichment cultures ranged between 10 and 60%. Most of the dominant OTUs other than methanotrophs were closely related to obligate or facultative methylotrophs, including *Methylophilus* spp., *Methyloversatilis* spp., and *Hyphomicrobium* spp. (Fig. S1). Previous studies suggested that methylotrophs utilize intermediate compounds of methane oxidation, such as methanol and formate released by methanotrophs (Krause *et al.*, 2017; Yu and Chistoserdova, 2017); therefore, the predominance of methylotrophs in our enrichment cultures is reasonable. However, it is important to note that the profiles of non-methanotrophic communities also completely differed depending on the supplemented REEs.

### Isolation of putative REE-dependent methanotrophs

**Fig. 2.**
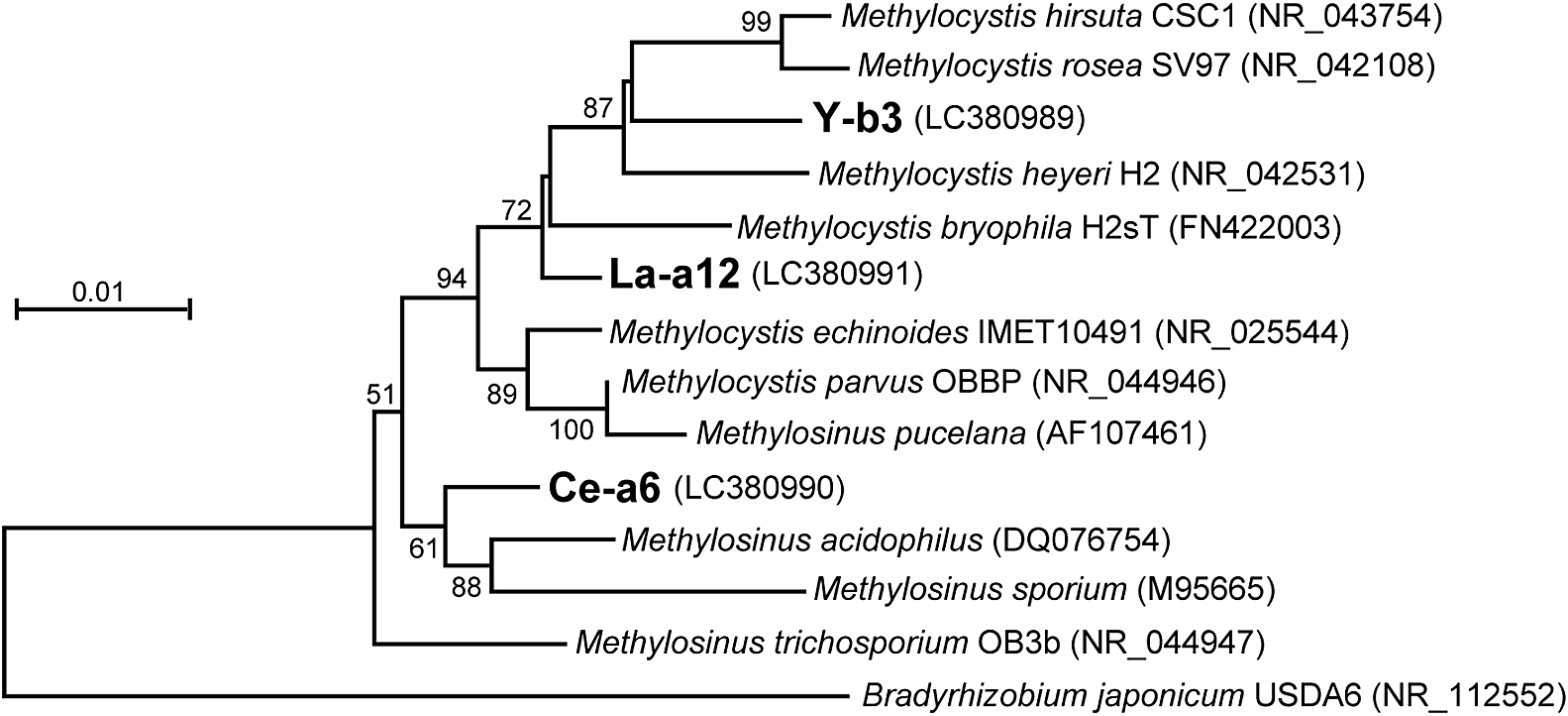
Phylogenetic tree based on 16S rRNA gene sequences of methanotrophs in the family *Methylosystaceae* and strains isolated in the present study. *Bradyrhizobium japonicum* was used as an outgroup. Bootstrap values (1,000 trials, only >50% are shown) are indicated at branching points. The scale bar indicates 1% sequence divergence. Accession numbers are given in parentheses.

We then attempted to isolate methanotrophs from enrichment cultures. Twenty-nine methanotrophic strains were isolated and classified into 3 phylotypes based on their partial 16S rRNA gene sequences with a cut-off value of 98% identity. Representatives of the three phylotypes, namely, strains Y-b3, La-a12, and Ce-a6, isolated from the +Y, +La, and +Ce enrichments, respectively, were subjected to phylogenetic analyses based on their nearly full-length 16S rRNA gene sequences. The phylogenetic analysis classified all isolates into the genus *Methylosinus* or *Methylocystis* in the family Methylocystaceae (Fig. 2). Furthermore, the sequences of strains Y-b3, La-a12, and Ce-a6 showed high identity (>98.7%) to the sequences of OTUs that dominated the corresponding enrichment cultures (OTUs OM-01, -03, and -04, respectively) (Table S2). Therefore, we successfully isolated methanotrophs corresponding to all OTUs recovered from the enrichment cultures, except for OTU OM-02, which dominated the +Sc enrichment.

### Growth properties of isolated methanotrophs

**Fig. 3.**
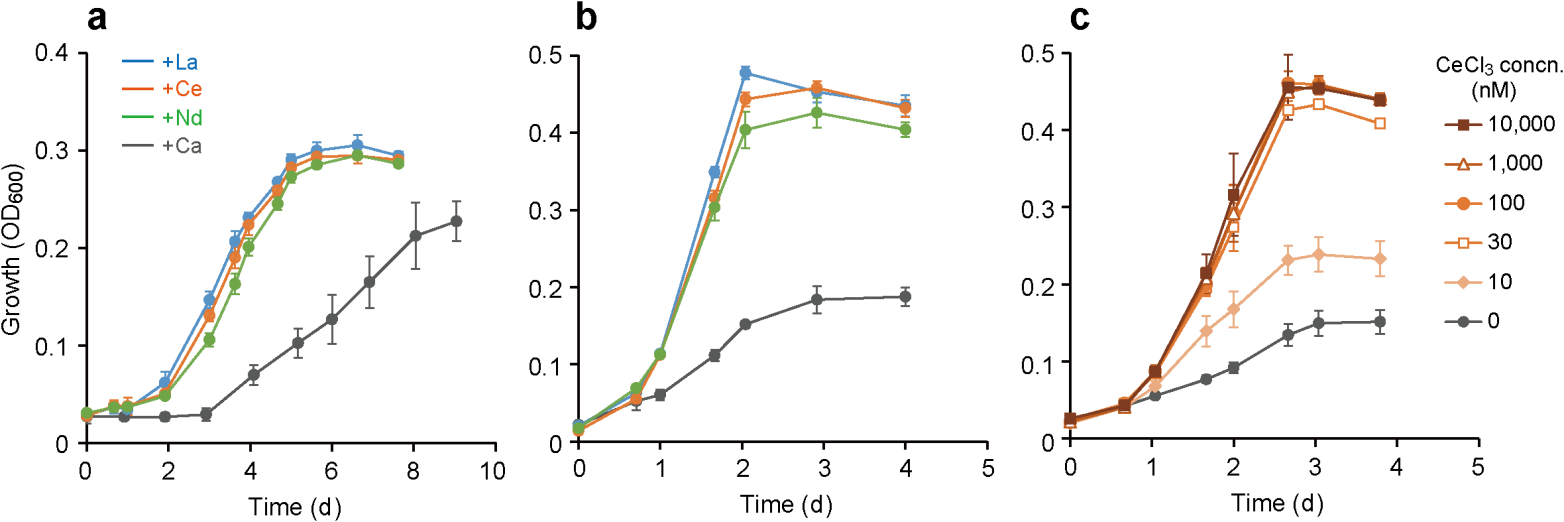
Effects of lanthanides on the growth of *Methylosinus* sp. Ce-a6. The growth of strain Ce-a6 with methane (a) (3rd subculture; see Fig. S3a for the 1st and 2nd subcultures) or methanol (b) as its carbon and energy source. Cultures were supplemented with 20 μM of chlorides of either Ca or a lanthanide (La, Ce, or Nd). (c) Growth of strain Ce-a6 on methanol with different concentrations of CeCl_3_. Data are presented as the means of three independent cultures, and error bars represent standard deviations.

Isolates were cultured in medium supplemented with chlorides of either Ca or a lanthanide (La, Ce, or Nd) to evaluate their lanthanide requirements. The growth of strains Y-b3 and La-a12 did not show any significant differences that were dependent on the addition of Ca or lanthanide chlorides (Fig. S2a and b). In contrast, strain Ce-a6 exhibited clear lanthanide dependency. The growth of strain Ce-a6 with methane as the substrate was significantly faster in the +lanthanide cultures (specific growth rate *μ*=0.60 to 0.71 d^–1^) than in the +Ca culture (*μ*=0.33 d^–1^) (Fig. S3a and b). A previous study reported that the lanthanide dependency of the Δ*mxaF* mutant of *M. trichosporium* OB3b became more conspicuous with repeated subcultures, possibly due to the alleviation of the carryover of lanthanides (Farhan Ul Haque *et al.*, 2016). Hence, each culture was further subjected to repeated subcultures. In the third subculture, the growth of strain Ce-a6 exhibited clearer lanthanide dependency; shorter lag phases and higher final cell densities were observed in the +lanthanide cultures (Fig. 3a). The difference between specific growth rates in the +lanthanide and +Ca cultures also increased (0.70 to 0.74 vs. 0.22 d^–1^, see Fig. S3b).

The lanthanide dependency of strain Ce-a6 was more clearly observed even without subculturing when methanol was used as the carbon and energy source (Fig. 3b). The specific growth rates of strain Ce-a6 were significantly higher in the +lanthanide cultures (*μ*=1.73 to 1.82 d^–1^) than in the +Ca culture (*μ*=0.89 d^–1^) (Fig. S3c). Therefore, we investigated specificities among REE species for the growth of Ce-a6 in methanol cultures (Fig. S3d). Among the REEs tested, only chlorides of La, Ce, and Nd supported the growth of strain Ce-a6, whereas chlorides of the non-lanthanide REEs (Sc and Y) and REEs heavier than Nd (samarium [Sm], gadolinium [Gd], Dy, and ytterbium [Yb]) did not exert growth-promoting effects. The observed specificity for REEs was similar to that of the lanthanide-dependent thermoacidophilic methanotroph; only relatively light lanthanides supported the growth of *M. fumariolicum* SolV (Pol *et al.*, 2014).

We also investigated the dose dependency of lanthanides on the growth of Ce-a6 in methanol cultures. The growth of strain Ce-a6 was significantly promoted by supplementation with only 10 nM CeCl_3_, and 30 nM was sufficient for its full growth (Fig. 3c). The full growth of strain Ce-a6 required a lower lanthanide concentration than that of the lanthanide-dependent strains reported to date, *i.e.*, the thermoacidophilic methanotroph *M. fumariolicum* SolV (>320 nM) (Pol *et al.*, 2014) and the mesophilic methylotroph *Novimethylophilus kurashikiensis* La2-4 (>1,000 nM) (Lv *et al.*, 2018).

We also evaluated the lanthanide requirements of the type strain of *M. sporium* (strain DSM17706^T^), the closest relative of strain Ce-a6 (Fig. 2). *M. sporium* DSM17706^T^ did not show lanthanide dependency; its growth was slightly better in the +Ca culture than in the +lanthanide cultures (Fig. S2c). It is important to note that the lanthanide dependencies of *M. sporium* DSM17706^T^ and strain Ce-a6 were markedly different despite their high phylogenetic similarity. The sequence identity of their 16S rRNA genes (99.0%) was markedly higher than the general criteria for classifying microbial species (97–98%). This result serves as a warning against a reliance on cultivation-independent analyses that are highly dependent on only 16S rRNA gene sequences.

### Utilization of insoluble lanthanides by strain Ce-a6

Concentrations of lanthanides in freshwater and seawater are generally sub-nanomolar (Elderfield *et al.*, 1990; Shiller *et al.*, 2017), which is one to two orders of magnitude lower than the minimum requirement for strain Ce-a6. These findings lead to the assumption that strain Ce-a6 has the ability to utilize insoluble lanthanides, *e.g.*, those contained in sand grains as insoluble oxide forms. To test this assumption, we performed culture experiments using insoluble Ce oxide (CeO_2_) as the source of lanthanide for strain Ce-a6.

**Fig. 4.**
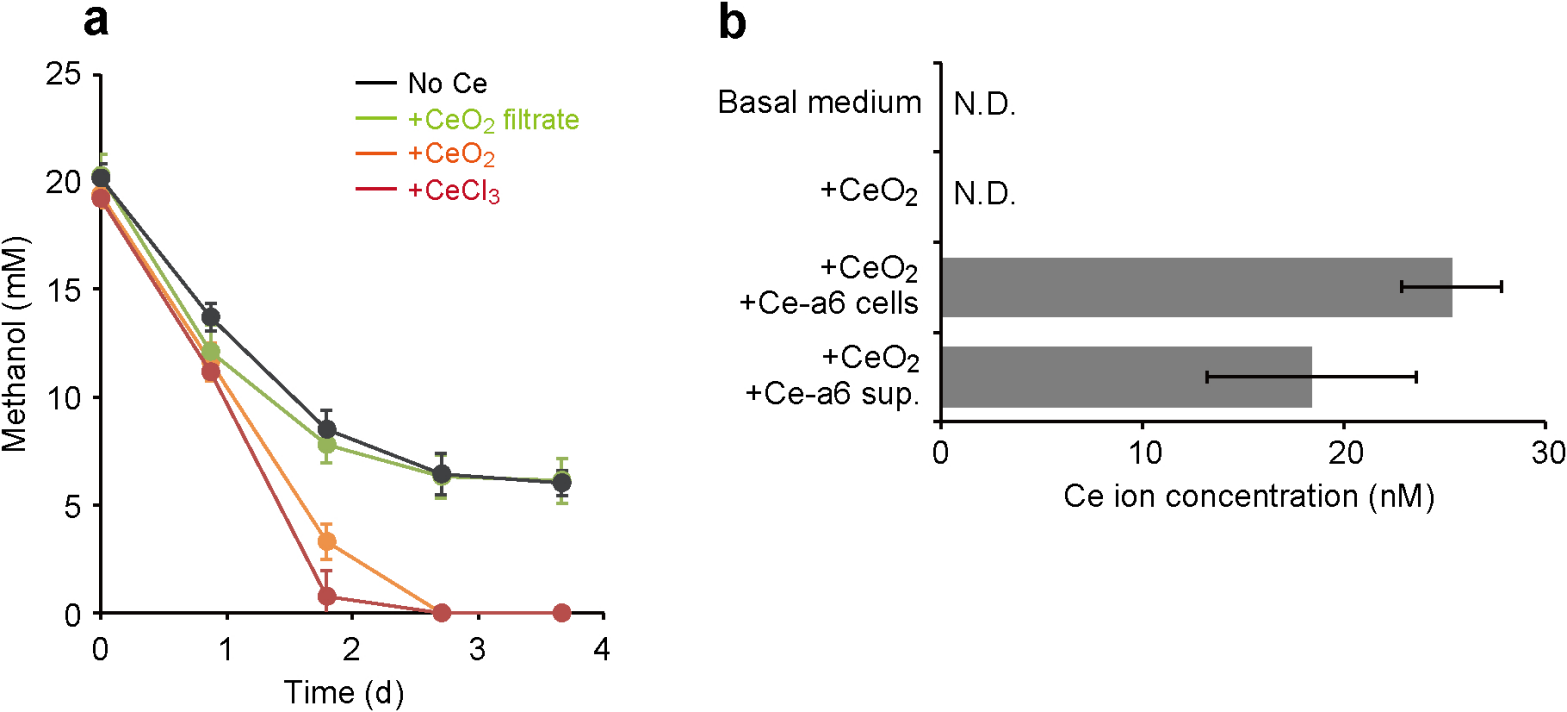
Solubilization and utilization of insoluble cerium oxide by *Methylosinus* sp. Ce-a6. (a) Growth of strain Ce-a6 in medium supplemented with different Ce sources. It is important to note that growth was assessed by the consumption of methanol because Ce oxide particles affected measurements of OD_600_ values (see Fig. S4). Black line; no addition, Green line; with the filtrate of the Ce oxide suspension, Orange line; with insoluble Ce oxide, and Red line; with soluble Ce chloride. (b) Dissolution of Ce ions from Ce oxide in the presence of growing cells of strain Ce-a6 (+Ce-a6 cells) or the filtrated spent medium of strain Ce-a6 (+Ce-a6 sup.). N.D.; not detected. Data are presented as the means of three independent cultures, and error bars represent standard deviations.

We initially evaluated the solubility of Ce oxide used in the present study. Ce oxide particles were suspended in inorganic basal medium, and its filtrate was prepared after 4 days incubation. The growth of Ce-a6 in the filtrate was the same level as that in control medium supplemented with no lanthanides (Fig. 4a and S4). The concentration of Ce ions in the filtrate assessed by ICP-OES was under the detection limit (<10 nM) (Fig. 4b). These results indicated that Ce oxide used in the present study was insoluble and did not supply the Ce ions required for the optimal growth of strain Ce-a6. In contrast, the growth of Ce-a6 was promoted in medium directly supplemented with Ce oxide particles, similar to the cultures supplemented with soluble Ce chloride (Fig. 4a and S4). Furthermore, 25.3±2.5 nM of Ce ions was detected from the filtrate of the strain Ce-a6 culture supplemented with Ce oxide (Fig. 4b). These results suggest that strain Ce-a6 has the ability to elute lanthanides from insoluble minerals, which may enable survival in circumneutral environments in which lanthanides only slightly dissolved.

Some microorganisms secrete specific chelating molecules to sequester essential metals in insoluble forms, *e.g.*, siderophores (Fe chelators produced by various bacteria) (Ahmed and Holmström, 2014) and methanobactin (Cu chelators produced by some methanotrophs) (Kim *et al.*, 2004). We hypothesized that strain Ce-a6 has the ability to produce and excrete chelating molecules that solubilize insoluble lanthanide minerals. The filtrated spent medium of strain Ce-a6 was supplied with Ce oxide particles, incubated for 4‍ ‍d, and subjected to an ICP-OES analysis (Fig. 4b). Ce oxide was solubilized by the filtrated spent medium and released 18.4±5.2 nM of Ce ions, suggesting that strain Ce-a6 secretes unknown chemical(s) that dissolve insoluble lanthanides. Martinez-Gomez *et al.* reported that the methanol-oxidizing bacterium *Methylobacterium extorquens* AM1 was capable of solubilizing lanthanides from particles of Neodymium magnet (2016); however, the underlying molecular mechanisms remain unknown. These findings indicate that at least some methane/methanol-oxidizing bacteria that harbor XoxF-MDH have special systems to utilize insoluble lanthanide minerals.

### The lanthanide-dependent methanotroph exclusively has a XoxF-MDH

To elucidate the molecular mechanisms underlying lanthanide dependency, we performed a draft genome analysis on strain Ce-a6 and *M. sporium* DSM17706^T^. The final assembly was based on 4,741‍ ‍Mb Illumina data and 169× input read coverage for strain Ce-a6, and 4,795‍ ‍Mb Illumina data and 160× input read coverage for *M. sporium* DSM17706^T^. The draft genome of strain Ce-a6 consists of 119 contigs with a total size of 4,099,638 bp and a 65% G+C content. Among 3,874 genes, there were 3,818 protein-coding genes, 52 tRNA genes, and 3 rRNA genes. The draft genome of *M. sporium* DSM17706^T^ harbored 114 contigs and 4,435,731 bp, with a 65% G+C content. Among 4,105 total predicted genes, 4,049 were protein-coding genes, 52 were tRNA genes, and 3 were rRNA genes.

**Fig. 5.**
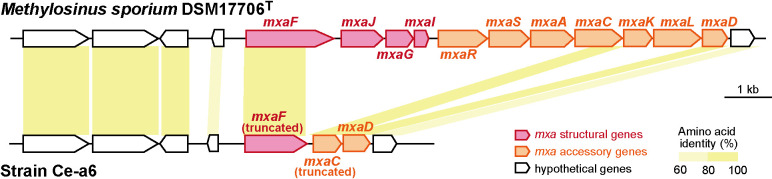
Organization of MxaF-MDH gene clusters in genomes of *M. sporium* DSM17706^T^ and strain Ce-a6.

The genome of *M. sporium* DSM17706^T^ contained one copy each of the XoxF- and MxaF-MDH gene clusters. The organization of gene clusters for XoxF- (*xoxFJG*) and MxaF-MDH (*mxaFJGI* and *mxaRSACKLD* for structural and accessory proteins, respectively) were well conserved with the other alphaproteobacterial methanotrophs (Keltjens *et al.*, 2014). The genome of strain Ce-a6 contained the gene cluster for XoxF-MDH with the same organization as, and high amino acid identities (90% to 97%) to, the XoxF-MDH cluster of *M. sporium* DSM17706^T^ (Fig. S5a). Strain Ce-a6 also harbored an *mxaF*-like gene (Ce-*mxaF*) in its genome. However, gene organization around Ce-*mxaF* markedly differed from that around *mxaF* of *M. sporium* DSM17706^T^ (Ms-*mxaF*) (Fig. 5). Ce-*mxaF* (440 amino acids [aa]) was markedly shorter than Ms-*mxaF* (621 aa) and *mxaF* of other alphaproteobacterial methanotrophs (621–634 aa). Ce-*mxaF* showed high amino acid identity (92%) only to the N-terminal region of Ms-*mxaF*. These results clearly suggest the truncation of the C-terminal region of Ce-*mxaF*. The gene next to Ce-*mxaF* showed high amino acid identity (92%) to the C-terminal region of Ms-*mxaC* located approximately 6‍ ‍kb downstream of Ms-*mxaF* (Fig. 5). Furthermore, a PCR analysis using degenerate primers targeting proteobacterial *mxaF* (Neufeld *et al.*, 2007) showed no amplification from the genomic DNA of strain Ce-a6 (Fig. S6). This result ruled out the possibility of the presence of integral *mxaF* gene(s) in the region that could not be examined by the draft genome analysis. Collectively, these results revealed a large-scale deletion (>6‍ ‍kb) around the MxaF-MDH cluster region of the strain Ce-a6 genome, which may cause its lanthanide dependency.

The expression levels of *xoxF* and truncated *mxaF* of strain Ce-a6 in the presence and absence of CaCl_2_ and CeCl_3_ were measured by quantitative RT-PCR (Fig. S7) to confirm that XoxF-MDH is the only MDH functioning in strain Ce-a6. *xoxF* was strongly and constitutively expressed, irrespective of the availability of Ca and Ce ions (expression levels of 0.29–0.50, normalized by the expression of the *pmoA1* gene). On the other hand, the expression of the truncated *mxaF* was low under all conditions tested (expression levels of 0.015–0.019). The expression pattern observed was completely different from that of methanotrophs harboring both MxaF- and XoxF-MDHs; the expression levels of MxaF- and XoxF-MDHs were suppressed and elevated, respectively, in the presence of lanthanides (Farhan Ul Haque *et al.*, 2015; Chistoserdova, 2016; Krause *et al.*, 2017). These gene expression results suggested that strain Ce-a6 exclusively utilizes XoxF-MDH, but not MxaF-MDH, irrespective of the availability of lanthanides.

Strain Ce-a6 showed significant growth in the absence of lanthanides (*e.g.*, the +Ca culture in Fig. 3) despite lacking MxaF-MDH. This may be because XoxF-MDHs appear to have very weak catalytic activity even when it coordinates Ca^2+^ in its active center, which was suggested for XoxF-MDH purified from *M. extorquens* AM1 (Schmidt *et al.*, 2010) and *Candidatus* Methylomirabilis oxyfera (Wu *et al.*, 2015). It is also possible that the alcohol dehydrogenases of strain Ce-a6, which is originally utilized for the oxidation of alcohols other than methanol, non-specifically oxidize methanol. Further biochemical analyses (*e.g.*, measurements of the methanol-oxidizing activities of XoxF-MDH and/or alcohol dehydrogenases) and genetic studies (*e.g*., construction of a *xoxF*-disrupted mutant) on strain Ce-a6 are required to prove these assumptions.

## Conclusion

This is the first study to report the isolation of mesophilic and neutralophilic methanotrophs that require lanthanides for optimal growth. The results obtained suggest that the availability of lanthanides is one of the important determinants of the structure and activity of methanotrophic communities in natural environments. Culture-independent analyses have indicated the presence of thousands of as-yet uncultured “unknown methanotrophs” in natural environments (Knief, 2015). The present study suggests that at least some of these have unusual lanthanide utilization characteristics, and indicates that “just adding lanthanides” (Skovran and Martinez-Gomez, 2015) enables the isolation of phylogenetically and functionally novel methanotrophs.

## Supplementary Material

Supplementary Material
