# Supplementary material for: Isolation and Genomic Characterization of a Proteobacterial Methanotroph Requiring Lanthanides
Source: Microbes Environ. 2020 Feb 8;35(1):ME19128. doi: 10.1264/jsme2.ME19128 (PMC7104280; doi:10.1264/jsme2.ME19128)
Supplement: Supplementary file 1 — Supplementary Material [file 35_19128_s1.pdf]

Supplementary Information

Isolation and genomic characterization of a proteobacterial methanotroph requiring lanthanides

Souichiro Kato[1,2,*], Motoko Takashino[1], Kensuke Igarashi[1], Wataru Kitagawa[1,2,3]

[1]Bioproduction Research Institute, National Institute of Advanced Industrial Science and Technology (AIST), Sapporo, Japan; [2]Division of Applied Bioscience, Graduate School of Agriculture, Hokkaido University, Sapporo, Japan; [3]Computational Bio Big Data Open Innovation Laboratory (CBBD-OIL), AIST, Sapporo, Japan.

*Address correspondence to Souichiro Kato, s.katou@aist.go.jp,

Figs. S1-7

Table S1-2

Fig. S1. Phylogenetic distribution of operational taxonomic units (OTUs) in enrichment cultures. Clone library analysis targeting the 16S rRNA gene was conducted for methanotrophic communities enriched from pond sediment in mineral medium supplemented with 20 µM of chlorides of either Ca, non-lanthanide REEs (Sc and Y) or lanthanides (La, Ce, Nd, or Dy). The relative abundance patterns of the dominant OTUs (>5% in at least one condition) were subjected to the hierarchical cluster analysis and heatmap drawing using a Morpheus software (https://software.broadinstitute.org/morpheus). OTU numbers and their closest relatives (sequence identity, %) are shown in the legend. OTUs classified into known methanotrophs and methylotrophs are highlighted in red and blue, respectively.

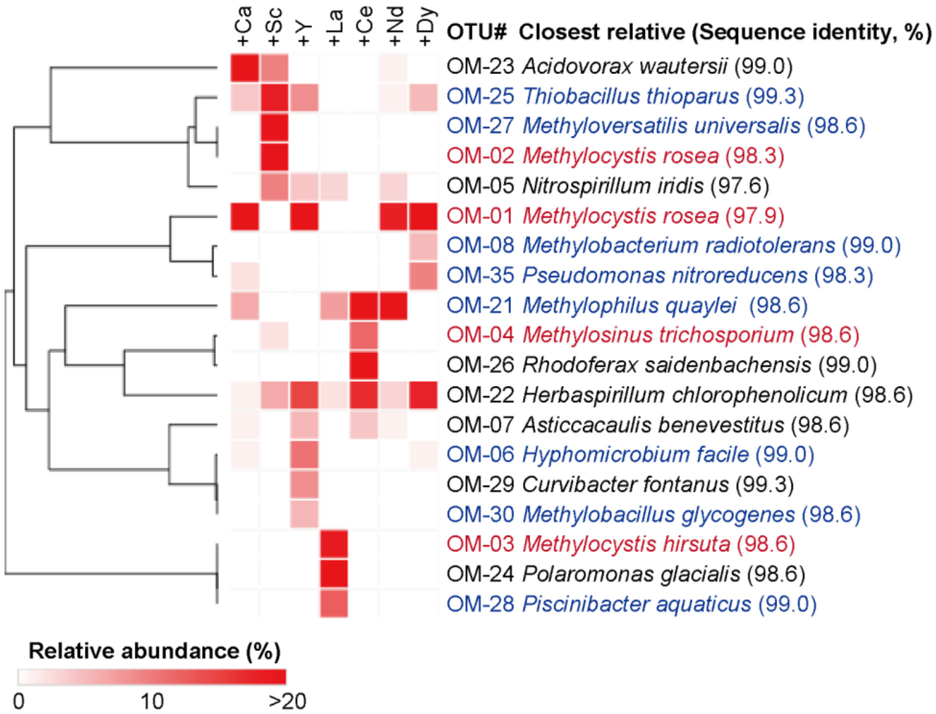

Fig. S2. Effects of lanthanides on growth of methanotrophic isolates and *Methylosinus sporium*. *Methylocystis* sp. Y-b3 (a), *Methylocystis* sp. La-a12 (b), and *Methylosinus sporium* DSM 17706^T (c) were cultured in medium supplemented with 20 µM of chlorides of either Ca or a lanthanide (La, Ce, or Nd) with methane as the carbon and energy source. Data are presented as the means of three independent cultures, and error bars represent standard deviations.

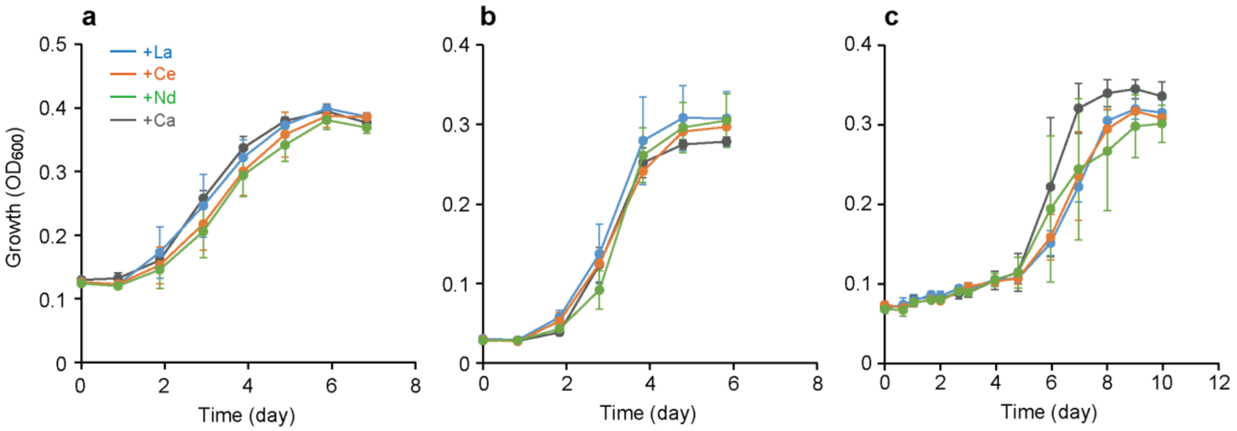

Fig. S3. Effects of rare earth elements (REEs) on growth of *Methylosinus* sp. Ce-a6. (a) Growth of strain Ce-a6 with methane as the carbon and energy source and 20 µM of chlorides of either Ca or a lanthanide (La, Ce, or Nd). Arrowheads indicate the subculture start-times. (b) Specific growth rates of strain Ce-a6 in methane cultures calculated from the data in (a). (c) Specific growth rates of strain Ce-a6 in methanol cultures calculated from the data in Fig. 3b. (d) Effects of different REE chlorides on the growth of strain Ce-a6 in methanol cultures. Data are presented as the means of three independent cultures, and error bars represent standard deviations.

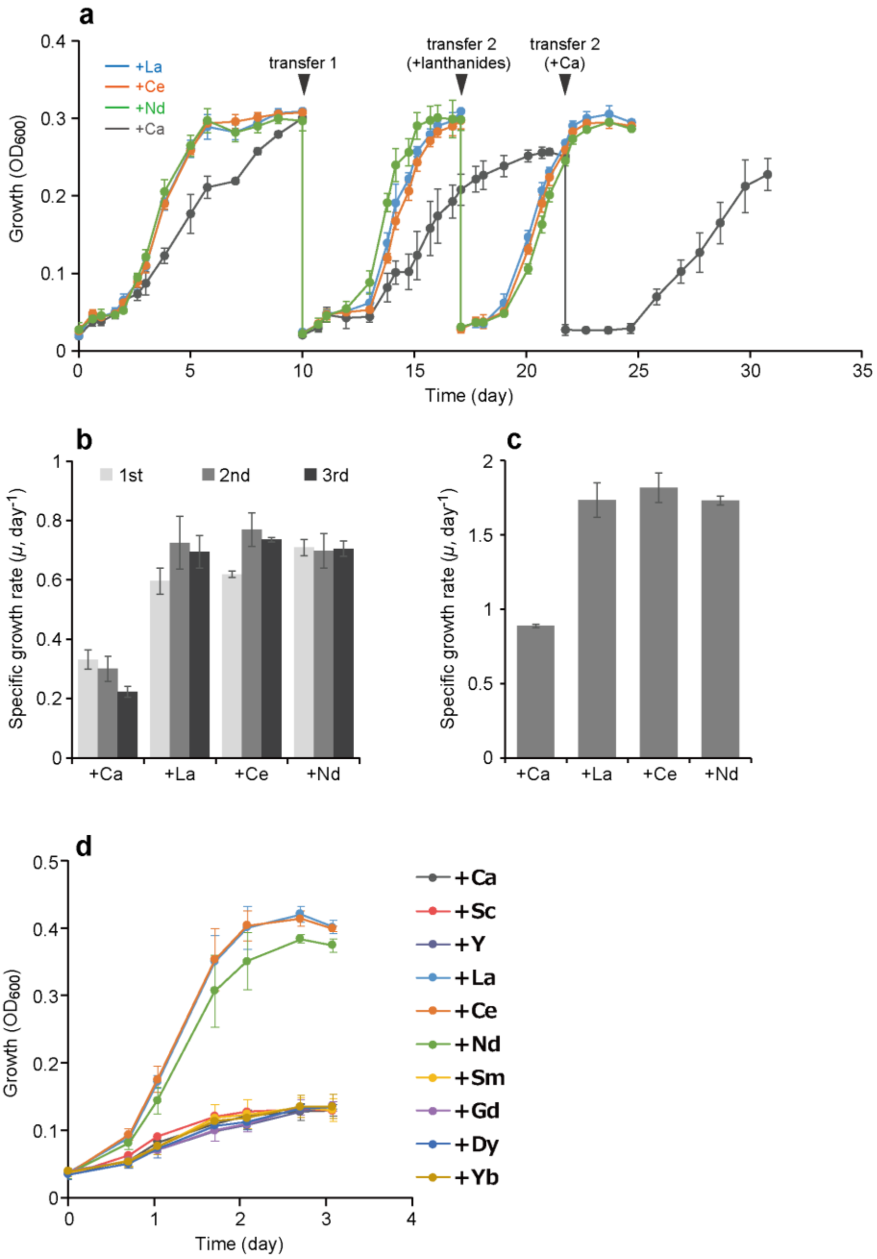

Fig. S4. Growth of strain Ce-a6 in the medium supplemented with different Ce sources. Black line; no addition, Green line; with filtrate of Ce oxide suspension, Orange line; with insoluble Ce oxide, and Red line; with soluble Ce chloride.

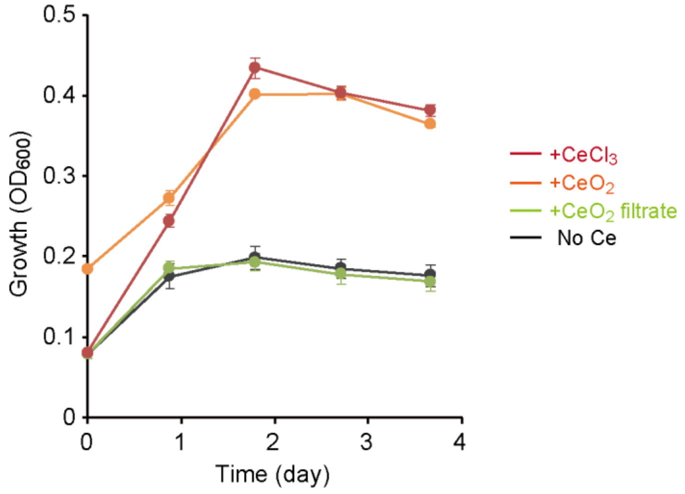

Fig. S5. Comparison of the arrangement of genes around the gene clusters of methanol dehydrogenases (MDHs) in the genomes of *Methylosinus sporium* (*Ms*) and strain Ce-a6 (Ce)**.** The gene cluster for Xox-MDH was well conserved between *Methylosinus sporium* and strain Ce-a6. The comparative genome analysis was conducted using the GenomeMatcher program (Ohtsubo *et al.*, 2008). Information of the gene annotations are also shown.

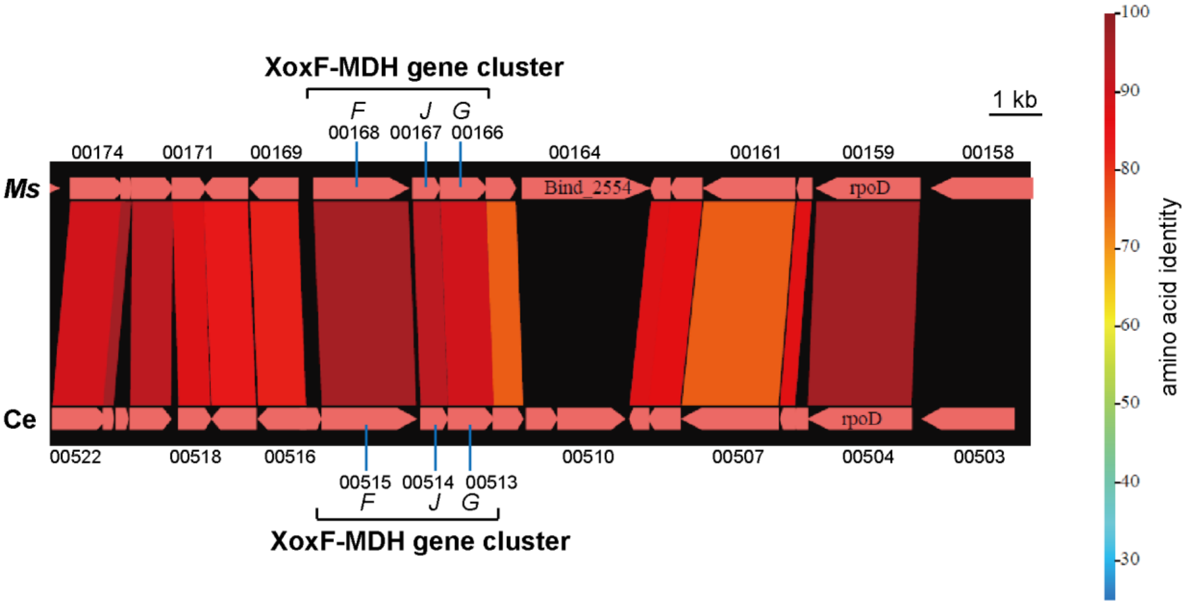

Gene Annotations for *Ms*

Gene ID	Annotation
Msp_00174	Glycine oxidase
Msp_00173	hypothetical protein
Msp_00172	Thiazole synthase
Msp_00171	Thiamine-phosphate synthase
Msp_00170	hypothetical protein
Msp_00169	hypothetical protein
**Msp_00168**	**Methanol dehydrogenase subunit 1**
**Msp_00167**	**Cytochrome c-553I**
**Msp_00166**	**hypothetical protein**
Msp_00165	hypothetical protein
Msp_00164	hypothetical protein
Msp_00163	hypothetical protein
Msp_00162	hypothetical protein
Msp_00161	Type IIS restriction enzyme Eco57I
Msp_00160	hypothetical protein
Msp_00159	RNA polymerase sigma factor RpoD
Msp_00158	DNA primase

Gene Annotations for Ce

Gene ID	Annotation
Ce-A6_00522	Glycine oxidase
Ce-A6_00521	hypothetical protein
Ce-A6_00520	hypothetical protein
Ce-A6_00519	Thiazole synthase
Ce-A6_00518	Thiamine-phosphate synthase
Ce-A6_00517	hypothetical protein
Ce-A6_00516	hypothetical protein
**Ce-A6_00515**	**Methanol dehydrogenase subunit 1**
**Ce-A6_00514**	**Cytochrome c-553I**
**Ce-A6_00513**	**hypothetical protein**
Ce-A6_00512	hypothetical protein
Ce-A6_00511	hypothetical protein
Ce-A6_00510	4'-demethylrebeccamycin synthase
Ce-A6_00509	hypothetical protein
Ce-A6_00508	hypothetical protein
Ce-A6_00507	Type IIS restriction enzyme Eco57I
Ce-A6_00506	hypothetical protein
Ce-A6_00505	hypothetical protein
Ce-A6_00504	RNA polymerase sigma factor RpoD
Ce-A6_00503	DNA primase

Fig. S6. Detection of the genes for methanol dehydrogenases (MDHs). The structural genes for two MDHs (*mxaF* for Mxa-MDH and *xoxF* for Xox-MDH) were amplified by PCR from the genomes of strain Ce-a6 and *Methylosinus sporium* (M.s.) using degenerate primers. *mxaF* primer pair: 1003f (5′-GCG GCA CCA ACT GGG GCT GGT-3′) and 1555r (5′-CAT GAA BGG CTC CCA RTC CAT-3′) (Neufeld *et al.*, 2007); expected size, 559 bp. Note that the faint band at a position larger than the predicted size amplified from the strain Ce-a6 genome was confirmed as a misamplification product derived from the truncated *mxaF* by sequencing analysis. *xoxF* primer pair: *xoxF5*-1720f (5′-GAY GAV TGG GAY TWY GAC GG-3′) and *xoxF5*-2144r (5′-GGY TCV TAR TCC ATR CA-3′) (Taubert *et al.*, 2015); expected size, 409 bp. M: 100-bp marker. Amplification conditions were as follows: an initial denaturation at 94 °C for 10 min, followed by a 30-cycle amplification consisting of 94 °C for 30 s, 55 °C for 30 s, and 72 °C for 1 min, with a final elongation at 72 °C for 10 min.

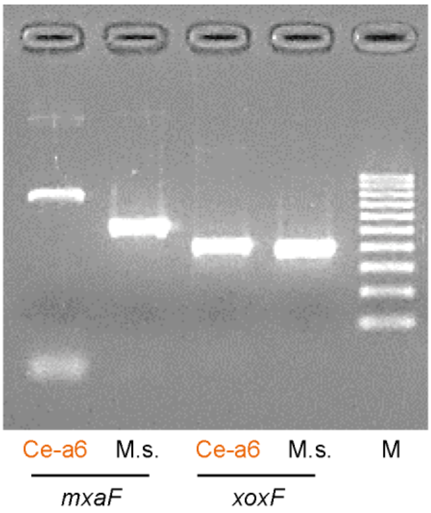

Fig. S7. Expression of the genes for XoxF- and MxaF-MDH in strain Ce-a6. Strain Ce-a6 was cultured in the presence and absence of $CaCl_2$ and $CeCl_3$ (20 μM each) with methane as the carbon and energy source. Total RNA was extracted from the log phase cells and was subjected to the quantitative RT-PCR analysis targeting the *xoxF* and the truncated *mxaF* genes. The expression levels were normalized by expression of the *pmoA1* gene. Data are presented as the means of three independent cultures, and error bars represent standard deviations.

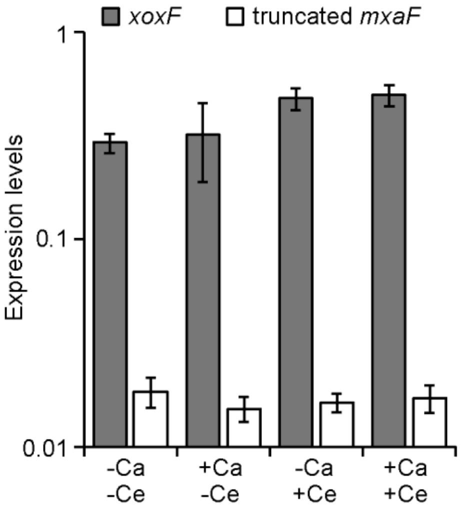

Table S1. The bacterial phylotypes detected in the enrichment cultures.

OTU number	Phylogenetic group	Closest relative			Number of clones						
		Strain name	Sequence identity (%)	Accession No.	+Ca	+Y	+Sc	+La	+Ce	+Nd	+Dy
OM-01	Alphaproteobacteria	Methylocystis rosea strain B-3422	97.9	LT220849.1	40		32			17	56
OM-02	Alphaproteobacteria	Methylocystis rosea strain B-3422	98.3	LT220849.1		20					
OM-03	Alphaproteobacteria	Methylocystis hirsuta partial strain B-3183	98.6	LT220847.1				18			
OM-04	Alphaproteobacteria	Methylosinus trichosporium OB3b	98.6	CP023737.1		2			11		
OM-05	Alphaproteobacteria	Nitrospirillum iridis strain YC6995	97.6	NR_146015.1		9	4	3		3	
OM-06	Alphaproteobacteria	Hyphomicrobium facile partial 16S rRNA gene, strain IFAM B522	99.0	Y14312.1	1		10				1
OM-07	Alphaproteobacteria	Asticcacaulis benevestitus strain Z-0023	98.6	NR_042433.1	1		5		4	1	
OM-08	Alphaproteobacteria	Methylobacterium radiotolerans JCM 2831	99.0	LT985974.1							5
OM-09	Alphaproteobacteria	Prosthecomicrobium hirschii strain 16	99.0	NR_104906.1	1	2			1		
OM-10	Alphaproteobacteria	Bradyrhizobium japonicum strain L16	99.3	KY412844.1				2		1	
OM-11	Alphaproteobacteria	Mesorhizobium japonicum strain Opo-242	99.0	MF661788.1			2				
OM-12	Alphaproteobacteria	Azospirillum picis strain R2A6.5-8	98.3	MF062684.1		2					
OM-13	Alphaproteobacteria	Phenylobacterium sp. URHA0022	98.3	LN876295.1	1			1			
OM-14	Alphaproteobacteria	Sphingomonas jaspsi strain NBRC 102120	99.0	NR_114034.1				1			
OM-15	Alphaproteobacteria	Azospirillum lipoferum strain KHAz	99.0	MG196053.1							1
OM-16	Alphaproteobacteria	Arsenicitalea sp. strain 14_AG719_1	98.6	MG232296.1					1		
OM-17	Alphaproteobacteria	Sphingomonas lutea strain JS5	98.6	NR_153746.1					1		
OM-18	Alphaproteobacteria	Sphingomonas kaistensis strain H11	98.6	JQ579647.1		1					
OM-19	Alphaproteobacteria	Caulobacter vibrioides strain CB1	99.0	CP023314.2			1				
OM-20	Alphaproteobacteria	Agrobacterium tumefaciens strain HAOB_M11	99.3	MH064220.1			1				
OM-21	Betaproteobacteria	Methylophilus quaylei strain MT	98.6	AY772089.2	6			7	32	62	
OM-22	Betaproteobacteria	Herbaspirillum chlorophenolicum strain YHNT18	98.6	MG571754.1	1	6	14	2	16	3	17
OM-23	Betaproteobacteria	Acidovorax wautersii strain PYGV-2	99.0	MF062564.1	35	9				1	
OM-24	Betaproteobacteria	Polaromonas glacialis strain BMP50	98.6	MG952607.1				45			
OM-25	Betaproteobacteria	Thiobacillus thioparus strain NZ	99.3	KC542801.1	4	17	8			1	5
OM-26	Betaproteobacteria	Rhodoferax saidenbachensis strain OX0321	99.0	MG576020.1					27		
OM-27	Betaproteobacteria	Methyloversatilis universalis strain B3	98.6	MG757548.1		22					
OM-28	Betaproteobacteria	Piscinibacter aquaticus strain BT19	99.0	KY284077.1				12			
OM-29	Betaproteobacteria	Curvibacter fontanus strain: AQ11	99.3	AB120965.1			8				
OM-30	Betaproteobacteria	Methylobacillus glycogenes partial 16S rRNA gene, strain DSM 462	98.6	LN998177.1			5				
OM-31	Betaproteobacteria	Sulfuriferula multivorans	98.6	LC005593.1		2					
OM-32	Betaproteobacteria	Pelomonas puraquae strain AT-91	98.6	KF817703.1						2	
OM-33	Betaproteobacteria	Cupriavidus taiwanensis strain ame16	98.6	MF680648.1	1						
OM-34	Betaproteobacteria	Aquabacterium parvum strain B6	98.6	NR_024874.1						1	
OM-35	Gammaproteobacteria	Pseudomonas nitroreducens strain BA010	98.3	KY616635.1	2						9
OM-36	Gammaproteobacteria	Pseudomonas mendocina strain HCB6	99.0	KF534475.1			1			2	
OM-37	Gammaproteobacteria	Nevskia ramosa strain: MAFF 211643	99.3	AB518684.1	1						
OM-38	Gammaproteobacteria	Thermomonas brevis strain Kh.AA.W5	98.6	KM199275.1	1						
OM-39	Bacteroidetes	Heliimonas saccharivorans strain L2-4	99.0	NR_135701.1		2		1			
OM-40	Bacteroidetes	Nubsella zeaxanthinifaciens strain M8	99.3	KF358271.1			2			1	
OM-41	Bacteroidetes	Chitinophaga sancti strain HAMBI 1988	99.3	LT899972.1				2			1
OM-42	Bacteroidetes	Sediminibacterium salmoneum isolate OTU-c67	95.5	KJ147078.1			1	1			
OM-43	Bacteroidetes	Mucilaginibacter oryzae strain: NBRC 106410	99.0	AB682426.1					1		
OM-44	Bacteroidetes	Algoriphagus sp. JJ018	99.0	JQ033987.1	1						
OM-45	Verrucomicrobia	Opitutus sp. VeSm13	96.9	X99392.1				1	1		1
				Total clone number	96	94	94	96	95	95	96

Table S2. Summary of phylogenetic analysis of three methanotroph strains isolated from enrichment cultures.

Isolate	Closest relative (identity, %)	Closest OTU (identity, %)
Y-b3	*Methylocystis rosea* strain SV97 (97.9)	OM-01 (98.7)
La-a12	*Methylocystis echinoides* strain IMET 10491 (98.9)	OM-03 (98.7)
Ce-a6	*Methylosinus sporium* strain SK13 (99.0)	OM-04 (99.1)

References for supplementary data

Costello, A.M., and M.E. Lidstrom. 1999. Molecular characterization of functional and phylogenetic genes from natural populations of methanotrophs in lake sediments. Appl. Environ. Microbiol. 65:5066–5074.

Ohtsubo, Y., W. Ikeda-Ohtsubo, Y. Nagata, and M. Tsuda. 2008. GenomeMatcher: a graphical user interface for DNA sequence comparison. BMC Bioinformatics. 9:376.

Taubert, M., C. Grob, A.M. Howat, O.J. Burns, J.L. Dixon, Y. Chen, and J.C. Murrell. 2015. XoxF encoding an alternative methanol dehydrogenase is widespread in coastal marine environments. Environ. Microbiol. 17:3937–3948.
